# Towards a set of competencies in palliative care nursing in Spain: what’s getting in the way of consensus?

**DOI:** 10.1186/s12904-024-01359-w

**Published:** 2024-02-14

**Authors:** Lourdes Guanter-Peris, Eulàlia Alburquerque-Medina, Montserrat Solà-Pola, Margarida Pla

**Affiliations:** 1 Catalan Institute of Oncology (ICO), Hospital Duran I Reynals, Avinguda de La Gran Via de L’Hospitalet,199-203, 08908 Barcelona, L’Hospitalet de Llobregat Spain; 2 Faculty of Nursing, University of Barcelona, S/N Feixa LLarga, Pavelló de Govern 3a Planta, 08907 Barcelona, L’Hospitalet de Llobregat Spain

**Keywords:** Professional competencies, Palliative care, Nursing experts, Framework

## Abstract

**Background:**

Spain currently lacks a competency framework for palliative care nursing. Having such a framework would help to advance this field in academic, governmental, and health management contexts. In phase I of a mixed-methods sequential study, we collected quantitative data, proposing 98 competencies to a sample of palliative care nurses. They accepted 62 of them and rejected 36.

**Methods:**

Phase II is a qualitative phase in which we used consensus techniques with two modified nominal groups to interpret the quantitative findings with the objective of understanding of why the 36 competencies had been rejected. Twenty nurses from different areas of palliative care (direct care, teaching, management, research) participated. We conducted a thematic analysis using NVivo12 to identify meaning units and group them into larger thematic categories.

**Results:**

Participants attributed the lack of consensus on the 36 competencies to four main reasons: the rejection of standardised nursing language, the context in which nurses carry out palliative care and other factors that are external to the care itself, the degree of specificity of the proposed competency (too little or too great), and the complexity of nursing care related to the end of life and/or death.

**Conclusions:**

Based on the results, we propose reparative actions, such as reformulating the competencies expressed in nursing terminology to describe them as specific behaviours and insisting on the participation of nurses in developing institutional policies and strategies so that competencies related to development, leadership and professional commitment can be implemented. It is essential ​​to promote greater consensus on the definition and levels of nursing intervention according to criteria of complexity and to advocate for adequate training, regulation, and accreditation of palliative care expert practice. Locally, understanding why the 36 competencies were rejected can help Spanish palliative care nurses reach a shared competency framework. More broadly, our consensus methodology and our findings regarding the causes for rejection may be useful to other countries that are in the process of formalising or reviewing their palliative care nursing model.

## Background

Contemporary phenomena, such as an ageing world population, the increase in noncommunicable diseases, and the emergence of novel viruses, recently highlighted by the COVID-19 pandemic, demonstrate that there is an immense demand for palliative care (PC), which is expected to double by 2060 [1]. In turn, the increasing complexity of health care at the end of life requires a competency framework that allows palliative care nurses to respond to the changing situations experienced by persons at the end of life. In Spain, unlike countries such as Ireland, Canada [2] and Australia [3], there is no regulation regarding competencies in PC.The variability of university training in nursing, as well as specific training from professional organisations and the fact that PC nursing [4] is not a recognised specialisation in Spain are factors that hinder the professional development of nurses in PC. Health policies developed in recent years have promoted the vigorous development of PC throughout Europe, but the lack of education and training opportunities have been repeatedly identified as obstacles to such development [5], as has the absence of an official specialisation process [6].

The vast majority of patients who die will be cared for by healthcare professionals for whom PC and the management of death is not their primary area of expertise [7]. The situations that nurses experience in PC are a continuous challenge for the development of their competencies in the relational, practical, and ethical dimensions. For this reason, they need knowledge, training, guidance and support to fulfil their role [8]. At the same time, nursing encompasses a range of dimensions that cannot be easily reduced to a mechanistic list of competencies. It encompasses a wide repertoire of skills that change according to the demands of each clinical specialisation in which nursing care is performed and therefore depends on the context [9].

The competency frameworks based on Virginia Henderson’s model, influential in Spain [10], along with the NANDA, NIC, and NOC languages [11], have been promoted by the General Council of Nursing in Spain since the 1990s [12], as well as by university nursing schools. The Council itself initiated the project for the Standardisation of Nursing Practice Interventions (NIPE in the original Spanish) in the national health system, driven by the Ministry of Health and Consumer Affairs in 2002, with the goal of establishing standardised methodology and common terminology. Currently, hospital working groups comprising 33 provincial nursing colleges are involved in the project. This team of over 400 professionals ensures the necessary consensus. However, from the outset, mechanisms were established to gather the largest number of professionals and institutions into the project. This implies that new professionals, institutions, and associations are continually being incorporated. Nevertheless, the use of common methodology and terminology is uneven and has not reached full implementation [10]. The factors involved, all of which are also associated with the development of the nursing role itself, are multifactorial.

In 2011, the Asociación Española de Enfermería en Cuidados Paliativos (AECPAL) initiated a line of research with the aim of developing a competency framework, based on the models of the International Council of Nurses [13], the Canadian Nurse Association [14] and the European Association of Palliative Care [15]. In 2013, AECPAL proposed 98 competencies grouped into three areas: ethics and law, delivery and management of care, and professional development. To seek professional consensus based on the proposed competency framework, a first phase of study was carried out in which 237 Spanish expert PC nurses were asked to what extent the proposed competencies fit the purview of PC nursing, concorded with actual PC nursing practice, and were important aspects of PC nursing [16]. The published results showed that of the 98 proposed competencies, 62 were accepted (defined as 75% of participants marking “yes” for purview and “high” or “very high” for concordance and importance). In contrast, 36 competencies were rejected (did not meet the 75% threshold for the three parameters) (Tables 1, 2, 3 and 4). All 98 proposed competencies reached the 75% threshold for importance.

**Table 1:** Count of competencies by area and 75% consensus reached (yes or no) for purview, concordance and importance

Competency area	Total	Purview		Concordance		Importance	
		Yes	No	Yes	No	Yes	No
Ethics and law	23	17	6	21	2	23	0
Delivery and management of care	55	51	4	43	12	55	0
Professional development	20	20	0	6	14	20	0
Total	98	88	10	70	28	98	0

Source: Authors et al., 2021 [16]

**Table 2:** Rejected competencies in ethics and law and parameters for which they failed to reach 75% consensus

		** < 75% consensus purview**	** < 75% consensus concordance**
**Responsibility**			
C 1.1	To respect the values, lifestyles and beliefs of the person during the care process. Adapting care, accordingly, even when opposed to the nurses’ own values	X	
C 1.2	To give support to the family in order to respect the values ​​and decisions of the person	X	
C 1.5^a^	To participate in the development of national and regional policies as well as guidelines in relation to the rights of the person at the end of life		X
**Ethical and legal rules**			
C 1.8	To be familiar with current regulations governing research processes and to ensure compliance, guaranteeing respect for the rights of persons who are research subjects	X	
**Ethical practice**			
C 1.11	To avoid the influence that the nurse’s own beliefs and values ​​may have on the delivery of care	X	
C 1.17	To maintain the principles of intimacy, confidentiality and dignity with the body after death	X	
C 1.18	To protect confidentiality and professional secrecy by recognizing that the owner of the information is the patient and information will only be shared with prior consent in the cases established by law	X	
C 1.23	To accompany the person to clarify their values, motives and consequences and to obtain specialized help if deemed necessary, in the request for assisted suicide, refusal of treatment or euthanasia		X

Source: Authors et al., 2021 [16]

^a^Competency was accidentally left off the nominal group discussion guide and therefore was not discussed by participants

**Table 3:** Rejected competencies in care delivery and management and parameters for which they failed to reach 75% consensus

		** < 75% consensus purview**	** < 75% consensus concordance**
**Essential principals surrounding the care plan**			
C2.10	To participate in and promote debate about innovations and changes in care for people in the process of advanced illness and the end of life		X
**Planning**			
C2.22	To define and prioritize nursing diagnoses with patient and family		X
C2.24	To define collaboration problems with the other professionals involved in the care process	X	
C2.29	To record the activation of specific techniques, protocols and procedures used, indicating the outcome criteria	X	X
**Evaluation**			
C2.34	To assess the results of activities outlined in the care plan activities, in relation to the planned objectives		X
C2.36	To use the results of assessments to deepen the individualization of the care plan		X
C2.37	To evaluate the results of the delegated activities, techniques, protocols and procedures used		X
**Therapeutic communication and interpersonal relationships**			
C2.46	To accompany the family after death by detecting specific needs in the grief process		X
**Safe environment, comprehensive care and resource management**			
C2.47	To prevent risk through early detection, communication and recording of security problems to the corresponding authorities	X	
C2.49	To develop criteria that allow assigning the most appropriate and capable nurse to provide care and attention, taking into account their knowledge and/or emotional response to the complexity of the situation		X
C2.50	To establish and maintain transdisciplinary and interdisciplinary relationships for decision-making that guarantee comprehensive care		X
C2.51	To use quality indicators and current or potential risk management adapted to the end-of-life situation		X
C2.54	To design specific care plans to support nurses at other levels of care in caring for people at the end of life		X
C2.55	To establish circuits and intervention criteria between the different levels of care involved in end-of-life care		X

Source: Authors et al., 2021 [16]

**Table 4:** Rejected competencies in professional development and the parameters for which they failed to reach 75% consensus

		** < 75% consensus purview**	** < 75% consensus concordance**
**Professional commitment**			
C3.3	To know and analyze the political and/or institutional situation related to the care needs of people at the end of life		X
C3.4	To implement the necessary changes at a professional, institutional and political level to improve care for people at the end of life		X
**Quality improvement**			
C3.7	To know, develop and apply quality indicators and standards of care plans for people at the end of life		X
C3.8	To participate in the evaluation processes and improvement of the quality of care for people at the end of life		X
C3.9	To incorporate the criteria of effectiveness and efficiency to guarantee the best care while optimizing available resources		X
C3.10	To generate resources to respond to specific care needs with quality criteria		X
C 3.11	To apply and disseminate the conclusions and proposals for improvement of the analysis of the results of the quality-of-care assessment		X
**Continuous training and teaching**			
C3.12	To lead the learning process for nurses in palliative care		X
C3.14	To participate in the detection of training needs and collaborate in the development, implementation and evaluation of educational programs in palliative care for all professionals in the health field		X
C3.15	To educate society about caring for people at the end of life		X
**Research**			
C3.17	To identify research priorities and establish feasible lines of research and develop research networks at the local, national and international level		X
C3.18	To consider the ethical issues of research with human beings who are vulnerable because they are at the end of life		X
C3.19	To acquire leadership, collaboration and promotion skills in palliative care research projects at the local, national and international levels		X
C3.20	To disseminate the results of research in palliative care		X

Source: Authors et al., 2021 [16]

The competencies in ethics and law involve actions in which the nurse is responsible for encouraging the patient to participate in their end-of-life process (keeping in mind their personhood and privacy) and for promoting the patient’s autonomy in decision-making within a framework of citizens’ rights. Table 2 demonstrates the rejected ethics and law competencies. For most of the rejected competencies in this area, the cause attributed by participants in the current phase was that it didn’t fall under the purview of PC.

The competencies surrounding delivery and management of care mostly involve creating and carrying out the individualised care plan along with the sick person and their family, while considering the person’s clinical situation of progressive fragility. Table 3 shows the rejected competencies in care delivery and management. For most of the rejected competencies in this area, the attributed cause was the lack of concordance with nursing practice in PC.

Finally, the competencies related to professional development concern nurses’ leadership in the development of PC in applying high-quality methods to improve care, participating in continuous training and teaching, and collecting scientific data to contribute to the improvement of the care for people at the end of life. Table 4 shows the rejected professional development competencies. For all the competencies in this area that didn’t reach consensus, the attributed cause was the lack of concordance with nursing practice in PC.

In the present phase of the study, our objective was to understand why 36 of the 98 competencies were rejected (that is, why they failed to reach 75% consensus across the three parameters). The next step will be to plan reparative actions, reword some of the competencies, and/or remove some of them from the AECPAL’s proposed competencies framework to achieve a shared competency model [17].

## Methods

### Design

We report on the second phase of a sequential explanatory mixed-methods study [18]. In Phase II, we used consensus techniques in two modified nominal groups, based on similar studies [19].

### Data collection

We presented to the participants the 36 rejected competencies [16] for discussion and consensus (Table 5). The principal investigator was the facilitator of both nominal groups. The first group met online and the second met in person.

**Table 5:** Order of discussion in both nominal groups (May and July 2018) and questions from the discussion about rejected competencies

The discussion took place in the following order: 1. Law and ethics competencies that did not reach consensus for purview (C1.1, C1.2, C1.8, C1.11, C1.17, C1.18) 2. Competencies in law and ethics that did not reach consensus for concordance (C1.5, C1.23) 3. Competencies in delivery and management of care that did not reach consensus for purview (C2.24, C2.47) 4. Competencies in delivery and management of care that did not reach consensus for concordance (C2.10, C2.22, C2.34, C2.36, C2.37, C2.46, C2.49, C2.50, C2.51, C2.54, C55) 5. Competency in delivery and management of care that did not reach consensus for purview or concordance (C2.29) 6. Competencies in professional development that did reach consensus for concordance (C3.3, C3.4, C3.7, C3.8, C3.9, C3.10, C3.11, C3.12, C3.14, C3.15, C3.17, C3.18, C3.19, C3.20)		
**Nominal Group**	**Format**	**Questions to start the discussion**
Nominal group 1 (May 2018)	online	1. Why do you think these competencies had low consensus? 2. Do you agree with that outcome? 3. Justify your answers individually 4. Reach consensus with the group on a joint answer
Nominal group 2 (June 2018)	in person	

The procedure involved the principal investigator, who also served as the moderator, conducting two systematic rounds of data collection with each of the nominal groups. Both rounds occurred in the same session for each group. In the first round, the moderator posed questions 1–3 for each set of competencies (see Table 5), giving individual participants the opportunity to respond. After listing the responses so that they were visible to participants, the moderator sought clarifications to avoid misinterpretations. In the second round, the objective was to reach an approximate 80% overall consensus [20] on the likely reasons for rejection within each competency group (question 4, see Table 5). To achieve this, the moderator again requested clarifications and reasoning for the responses while fostering debate. The moderator implemented interpretive adjustments with a hermeneutic orientation, such as asking participants to make their arguments explicit and avoiding offering her own interpretations.

### Participants

A total of 20 nurses participated; 9 in one nominal on-line group and 17 in the second nominal face-to-face group, 6 of whom were also in the first nominal group from different work areas (direct care, teaching, management, research) who met the following inclusion criteria: regional leader of the AECPAL board, > 5 years of professional nursing experience, and specific training. Because the target participants were regional leaders, they were known to us, and we contacted them directly by email. All 20 invitees agreed to participate, at which point we sent them the guidelines for participating in the nominal groups, the list of the competencies, and the dates and location, of the meetings (on-line or in person). Because participation in the prior phase of the study [15] was anonymous, we do not know for sure if any of the participants in the current phase also participated in the prior one. However, because they are leaders in the target population of PC nurses and are committed to the development of the competency model, it is likely that all of them did so.

### Data analysis

The analysis was conducted in two stages: 1) The groups were recorded and transcribed literally by LGP. 2) LGP and EAM performed inductive thematic analysis, identifying meaning units and grouping them into thematic categories (Table 6). Through a comparative analysis process, LGP and EAM independently coded and then reached a consensus on points of discrepancy, with the support of MS (see Acknowledgements), who also contributed to the analysis and interpretation of the data using NVivo12. Then, MS and MP reviewed the analysis and suggested alternative interpretations where necessary. Through this process, the team strengthened the analysis and made it explicit.

**Table 6:** The reasons for low consensus and competency areas

Reason for low consensus		Competency area		
**Theme**	**Subtheme**	**Ethics and law**	**Delivery and management of care**	**Professional development**
		Number of examples		
**1. Rejection of standardised nursing language (SNL)**	1.1 Low use of SNL in practice 1.2 Divergence between theory and practice 1.3 Little usefulness of SNL in practice	-	44	-
**2 Context**	2.1 Health policies and institutional strategic plans 2.2. The scarce presence of nurses in management positions 2.3. Multidisciplinarity	-	26	10
**3 Specificity**		27	-	2
**4 Complexity**		15	1	-

## Results

Of the 20 participants, 18 were women and two were men. The age range was 36 to 62 years old, with a median of 48. Eleven participants (55%) carried out direct care work exclusively, eight (40%) combined direct care, teaching, management, and research, and one (5%) was exclusively a researcher. The distribution of professional roles was similar in the two groups. We identified four alleged reasons for the weak consensus on the rejected competences: rejection of standard nursing language (SNL), context, specificity, and complexity (Table 6).

### Rejection of standardised nursing language (SNL)

The rejection of SNL is the reason attributed to all 14 rejected competencies in the delivery and management of care. Participants surmised that the rejection of these competencies was actually a rejection of the technical nursing terminologies (NANDA International, Nursing Interventions Classification, and Nursing Outcomes Classification, known jointly as NNN classification) [21]. The rejection of SNL fell into three sub-categories: low use of SNL in practice, divergence between theory and practice, and little usefulness of SNL in practice.

### Low use of SNL in practice

When asked about the competencies C2.10, C.2.22, C2.34, C.2.36, and C.2.37, one participant spoke about resistance to using nursing care plans and nursing diagnoses, which rely heavily on SNL.

*“I have the theory that there is a rejection by many people still with the issue of the nursing care plan, of the diagnoses and of all that process.” (Participant 6, Group 1)*

### Divergence between theory and practice

The participants reported that methodological competencies acquired in nursing studies (including SNL) aren’t reflected in nursing practice. When talking about C2.22, one participant responded as follows.

*“I believe that nursing, still, has not made the link that having methodology enhances the discipline and so you go through it at university, you’ve studied it, you’ve passed, and when you get to the work environment you return to do what they’ve been doing, and you copy others." (Participant 8, Group 2)*

### Little usefulness of SNL in practice

One participant, speaking about C2.37, reported that SNL is not a useful instrument and instead described it as having little efficacy and utility.

*“There’s also no evaluation in which you say, ‘This will help me improve.’” (Participant 19. Group 2)*

## Context

According to the participants, contextual elements external to the care itself, such as culture and health organisation, the work environment, economic resources, and multidisciplinarity are causes of the rejection of some competencies in delivery and management (C. 2.10, C. 2.22, C. 2.34, C. 2.36, C.2.37, C. 2.46, C. 2.49, C. 2.50, C. 2.51, C. 2.54, C. 2.55) and, of all of the rejected professional development competencies. We address the three most frequently cited contextual reasons here: health policies and institutional strategic plans, the scarce presence of nurses in management positions, and multidisciplinarity.

### Health policies and institutional strategic plans

According to the participants, health policies and strategic plans are decisive for the provision of care offered. The definition and prioritisation of nursing diagnoses with the patient and family, as stated in C2.22, the participants pointed out that health policies and management teams promote or hinder the use of the nursing process and even contribute to the scarce integration of SNL in practice. In turn, health management teams establish priorities based on the available resources, and therefore may not always facilitate the implementation of the nursing process. Speaking about C2.29, one participant remarked:

*“In my region, [I] have experienced a huge setback in the use of the nursing process. Enormous. To such an extent that when the main hospital was opened, approximately 6 years ago, the medical file [software] was purchased without the option of making a nursing care plan. With the issue of cutbacks, people stopped valuing systematically the completion and use of these methodologies...” (Participant 4, Group 1)*

The scarce presence of nurses in management positions limits nurses’ influence in institutional, political, and quality decisions, as this participant expressed when the group was discussing competencies C.3.3, C.3.4, C.3.7, and C.3.8.

*“How many nurses are involved in the design of regional palliative care programmes? Well, the percentage is not very high. And if nurses are not in the decision-making, then decisions will not be made with this vision.” (Participant 4, Group 2)*

### Multidisciplinarity

Finally, the multidisciplinary environments where PC takes place limit the autonomy and professional growth of nurses. When asked about C2.46, which is related to therapeutic communication and supporting the family, participants reported that PC nurses do not perform this task because it is “traditionally” handled by a social worker and/or psychologist.

*“In the contexts I know, it’s usually done by the psychologist or the primary care nurse. I think that is the reality, it should be ours, but maybe it’s not in practice, because of the organisation or whatever.” (*Participant 4*, Group 2)*

### Specificity

Participants reported that several ethics and law competencies were not specific to the purview of PC nurses, but rather were general to all nurses (C1.1, C1.2, C1.11, C1.17, C1.18). However, they reported that generalist nurses do not carry them out.

*“They are all basic [competencies]. What happens is that later you realise that when you work with nurses who are not in palliative care, as is my case, you realise that none of them do any of these, in practice.” (Participant 1. Group 1)*

Although participants surmised that these competencies had been rejected because they do not pertain exclusively to PC nursing, they also pointed out that C1.18 (related to confidentiality, privacy, dignity, and professional secrecy) was especially essential to the PC context.

*“It’s good that this competency is identified as an expert competency [that is, a competency of PC nursing], because it’s better to keep it in mind. Because, sometimes we handle information, I think you have to be more respectful of the information that everyone deals with within the family and with the patient” (Participant 3, Group 1)*

Finally, the ethics and law competency related to research (C. 1.8) is identified as specific to the field of the research nurse and not concordant with the practice of PC nurses broadly.

*“That one is specific [to PC nursing], yes I understand that, if you do not have a slightly more advanced training, that exceeds your competencies. In that case, I do think that it [the reason for rejection] is not because it’s cross-cutting, but that it’s too specific and I think they [the respondents in phase 1] understand that it’s not a specific competency.” (Participant 4, Group 1)*

The low consensus in this research competency reveals shortcomings in the area of research, as evidenced in the results for professional development competencies (Table 4).

### Complexity

The complexity of care emerges in a single ethics and law competency, C1.23, which deals with supporting patients who request the withdrawal of treatment, euthanasia, or assisted suicide. It was surprising to the participants—and to the research team—that this competency did not achieve consensus on the parameter of concordance, since it covers typical deliberative processes at the end of life. The participants initially attributed its rejection to poor wording. However, in the discussion that followed, what emerged was that the wording is clear but the scenario that it describes is complex. As defined by Codorniu and Tuca, the complexity of a clinical situation is the set of emergent characteristics of a case that in their particular interaction confer a special difficulty in decision-making, uncertainty in the outcome of the therapeutic intervention, and a consequent need to intensify specialised health intervention [22].

*“I guess people [the respondents] didn’t understand it right. They didn’t understand the statement. Let's see, supporting the person when someone asks you for euthanasia or assisted suicide or something, that support is something we should provide. But maybe someone understood that we were like agreeing to that request, right? And that’s why people didn’t agree.” (Participant 6, Group 1)*

*"Yes, even that [word] “support”, specify it a bit more... It’s to support, to look for the help that the person needs. Not sending that person to be euthanised, but in seeing what led them to make those decisions, helping them in the situation they find themselves in.” (Participant 2, Group 1)*

*“Assisted suicide and euthanasia if you express in instead as ‘shortening of life’ or ‘prolongation of life’, it’s not so rejected.” (Participant 17, Group 2)*

*“Surely they didn’t understand what that competency is, but I still don’t understand that it doesn’t concord with practice, because it happens to me all the time.” (Participant 9, Group 1)*

## Discussion

We asked a sample of leaders in PC nursing why they thought 36 of 98 proposed PC nursing competencies had been rejected by PC nurses in an earlier phase of the study. Our analysis of their responses revealed four main reasons: the rejection of SNL, contextual factors, specificity (too much or too little), and the complexity of end-of-life care.

The first purported reason for rejection was the rejection of SNL. Surprisingly, the participants sometimes identified aspects of general nursing common to daily practice as reasons why the PC competencies had been rejected. This tendency could be linked to the rejection of competencies expressed using SNL. The view that SNL is not very useful or well-integrated into clinical practice, as described by the participants, may be related to seeing SNL as a conceptual imposition [23]. This situation may be exacerbated by the chasm between the extensive use of SNL in academic settings [21] and its relative absence in care settings. Research reveals deficits and inaccuracies in nursing records and difficulties in the use of NNN, as well as controversies about its usefulness for representing care delivery and outcomes [24, 25]. In turn, in the study by Rios et al., nurses in primary care reported that these classifications are not very understandable, are difficult to use in care practice, and are not very useful, coinciding with our results [26]. However, AECPAL has opted to promote the use of SNL by developing a guide for standardised care plans [27] and incorporating a wearable infusion device into patient care [28]. These steps favour clinical judgement and decision-making based on the homogeneity and objectification of interventions and, by standardising them, enrich care plans. SNL is also key to nursing itself, can affect how nursing evolves in multidisciplinary environments such as palliative care. The review by Conolly et al. did not find a consensus on which SNL should be used in defining competency models in palliative care [29]. However, it is clear that SNL of some type must be used to describe specific nursing actions in order to make it possible to evaluate subsequently whether professionals’ follow them in practice, in line with the PIMAC project [30]. This project also sought consensus among experts to define competencies and create an evaluation instrument to identify the professional’s perception of her own competency level as well as her supervisor’s perception of her competency level. Undoubtedly, as Hokka et al. point out, nursing competencies in PC, especially those that are most relevant to each level of PC delivery, should be better described to improve development, education and practice [31].

The second reason why some competencies were rejected, according to the participants, was contextual factors that affect how nursing competencies can be carried out and determine the nursing model of care offered to patients and their families. As del Pino points out, in multidisciplinary contexts such as PC, it is especially important to use patient-centred care models that reflect nurses’ autonomous role, using SNL to improve the quality of care provided [32].

As the participants pointed out, health policies and institutional strategic plans tend not to empower nurses or place them in leadership roles, which in turn limits nurses’ influence on health planning in Spain. Proof of this was the non-concordance with the competencies related to development, leadership, and professional commitment. Hokka et al., however, found that if expert nurses develop their competencies related to professionalism and leadership, they can bring about changes in practice [31]. For the nursing perspective to have an impact on the quality of care, nurses must be present in management positions.

A growing number of people with advanced chronic health conditions and PC needs are dying without having their health and social needs met. This is reason enough to redefine traditional models of care with a view to focusing them on the person, rather than the disease [33]. Most health institutions are still governed by a disease-based model of care organised around medical specialties. This orientation generates a major source of ethical and professional conflicts in nursing practice.

In Spain, according to Codorniu et al., nursing care is not considered very important and receives little consideration at the policy level. Spain’s health system does not fully take into account the resources necessary to perform nursing care well, nor does it invest in evaluating its outcomes [34]. Economic and human resources are decisive for prioritising strategies for the development of nursing competencies. Nursing is consistently underfunded, as can be seen in the small number of nurses for each health area or specialisation, the scant investment in nursing research, and the lack of funding to promote a care model that includes SNL. Despite the findings, as institutions foster nurses’ clinical autonomy and support continuing education, nurses will undoubtedly have more opportunities to make decisions autonomously.

Nurses tend to cede leadership roles to other kinds of health professionals, an action that probably limits the quality of care that patients receive [32]. The Nursing Now movement proposes transition models that will facilitate changes in social attitudes towards nurses and the equalisation of functions, skills, knowledge and competencies [35]. Our results give visibility to the need to rethink how to integrate management skills by training nurses in leadership during their university studies and then helping them transition to leadership roles [36]. As we have argued, leadership is closely linked to ethics. In this sense, including leadership competencies in the model reflects this commitment from the international nursing code of ethics: “Nurses participate in professional governing bodies and associations so that the contribution of nursing is present in the planning and redesigning of health, academic and social policies” [37].

The third reason why competencies were purportedly rejected was problems related to the specificity of the proposed competencies; that is, some were too narrow or too broad, especially in the case of ethics. Paradoxically, the competencies that are most directly related to the principles of PC (C1.14 to help the person in a situation of advanced disease and at the end of life so that they can exercise their autonomy with their friends, family and care providers and C2.43 to create an intimate therapeutic context that stimulates communication) were considered broadly applicable to all nurses, as expressed in the nursing code of ethics [38]. These principles are present in all areas of care but are developed in greater depth in PC.

Care is a fundamental value on which PC nursing is based. According to this principle, the patient is treated as an individual and not as a condition or a disease [39, 40]. Several authors point out that experience, training in PC, and bioethical challenges are necessary for a nurse to develop the necessary confidence and skills to care for a person at the end of life [41]. Our findings suggest that, considering the complex life experiences that nurses in PC experience, greater consensus should be developed on the definition and criteria for care complexity and the different degrees of intervention according to the nurse’s expertise. This proposal is in line with Currow, who outlines the need to define levels of care, referral strategies, and resource allocation appropriate to each case [42].

At the same time, we must incorporate specialist and advanced practice nurses, given that patient care is increasingly complex [43]. Also according to Hokka et al., having staff members with insufficient skills in PC can hinder the learning of nursing students [44]. Therefore, expert nurses are key elements for the implementation of PC. Kennedy et al. demonstrated that PC, which emphasises a multi-professional approach, is undoubtedly an ideal environment to establish the functions of advanced practice nurses, who provide safe, effective, and person-centred care in the face of growing demands. They also argue that it is necessary to define and explain their functions, given the lack of clarity and regulatory frameworks in many countries [45].

According to Benner, because nurses acquire competencies over time, the competencies that they bring to bear in a given situation will vary with the degree of expertise that they have achieved [43]. Ethical competencies are required in each PC process since nurses interact regularly and meaningfully with people who are facing some of the most demanding and emotional moments of their lives [8]. Also, where the patient is cared for determines the nurse’s level of involvement. In this sense, while ethical competencies are crucial for all nurses, they are especially important in PC, where nurses interact intensely with patients.

Finally, the fourth reason that participants attributed to the rejection of competencies was complexity—referring to the emergence of processes that interact in complex systems [46]. Participants offered this reason for the rejection of the competency related to supporting the patient during the limitation of therapeutic effort, life support, euthanasia, and assisted suicide (C1.23) [47]. Undoubtedly, it is the request for assisted suicide or euthanasia—and not the rejection of treatment—that arouses debate. As Busquets says, it is a situation that implies a high fragility for the person and family due to the magnitude of the help they need and, in turn, is ethically complex for health professionals, including nurses [48].

Nurses have a code of ethics developed in 1973 and periodically updated by the International Council of Nurses (ICN) [38]. This code serves as a guide for resolving ethical issues that may arise in the practice of the profession in a way that respects human rights, including cultural rights, the right to life, freedom of choice, and the right to dignity and respect. In conjunction with the ethical and legal principles of PC focused on providing relief and comprehensive care, this framework guides the actions of the nursing community in Spain. Having a national strategy for PC [49] enables the promotion of the application of bioethical principles through recommendations for nursing care processes based on these principles and the current legislation in the different regions of Spain.

Even when PC professionals apply these international and national ethics strategies, the request for help to die can make PC professionals feel that they are not responding adequately to the needs of patients. This dilemma suggests a subsequent line of research to explore PC nurses’ self-efficacy and self-competence, described by Bandura as influencing the acquisition, development and achievement of competencies [50]. Undoubtedly, competency development must respond to the needs of patients since their requests act as signals that modulate professional behaviours [32]. Requesting help to die is not mutually exclusive with PC. In fact, Rosso et al. show that in some US states and Canada, between 81 and 92% of people who died by euthanasia had received specific PC [51]. At the time of data collection for phase I, there was no euthanasia law in Spain. The Euthanasia Regulation (Organic Law 3/2021) came into force on June 25, 2021, making it possible for people with terminal or seriously incapacitating conditions to request to end their life [48]. It remains to be seen how this new law will affect PC and the competencies of PC nurses. Finally, we cannot ignore that the attitude of PC nurses to death is influenced by external factors (society, culture, experiences) [52]. Therefore, it is essential to ensure that PC nurses have the appropriate training, professional guidance, and development.

## Limitations

The sample size was small. Additionally, the participants in this phase of study speculated on why the full set of respondents had rejected the 36 competencies in an earlier phase, which could be seen as a limitation because they were interpreting the responses of other people rather than providing explanations for their own opinions. However, the participants were drawn from the same population as in the first phase and were experts and leaders within it. Because these participants are precisely poised to make decisions about the PC competency model, having them interpret the rejection of the competencies by non-expert PC nurses is crucial for moving the competency model forward.

The Spanish Law of Euthanasia (Ley Orgánica 3/2021) [53] came into effect in 2021, meaning that some of participants’ answers about competency 1.23 might be different if data were collected now as opposed to in 2018. However, the rollout of the law has been slow and uneven, meaning that our analysis remains relevant. A follow-up study could examine how the new law has altered the landscape of PC competencies in Spain.

Data analysis was suspended due to COVID-19 because the principal investigator had to perform care and management functions at her hospital until the end of 2021, causing a delay in publication.

## Conclusions

Consensus in competency models is key, and this research offers a window onto what is getting in the way of consensus on a competencies model in PC in Spain. Further steps must be taken to achieve a shared competencies model. The proposed reparative actions—improving the wording of the competencies, promoting SNL at the policy level, encouraging nurses to take leadership roles, improving links between university studies and clinical practice, improving consensus about levels of intervention at the end of life, and improving consensus on the kinds of care provided by nurses with different levels of experience and expertise—require a joint effort by organisations and professionals. Additionally, giving visibility to the causes that hinder and/or influence the acceptance of the proposed competencies is already in itself a reparative measure.

Implementing a consensual competency model will favour the performance of PC nurses in multidisciplinary organisations, contributing to generate expert, comprehensive, personalised, high-quality care, which best fits the changing needs and expectations of society. ​​Our consensus method and our findings about the reasons that competencies were rejected may be useful for countries that wish to formalise or review their PC competency frameworks, since such frameworks must be dynamic documents subject to the changing needs of the population.

## Data Availability

The full proposed competency model is available on request or by consulting. The qualitative data are not available to other researchers because participants were promised confidentiality.
