# Correction: Towards a set of competencies in palliative care nursing in Spain: what’s getting in the way of consensus?

**DOI:** 10.1186/s12904-024-01397-4

**Published:** 2024-03-02

**Authors:** Lourdes Guanter‑Peris, Eulàlia Alburquerque‑Medina, Montserrat Solà‑Pola, Margarida Pla

**Affiliations:** 1 Catalan Institute of Oncology (ICO), Hospital Duran I Reynals, Avinguda de La Gran Via de L’Hospitalet, 199-203, Barcelona, L’Hospitalet de Llobregat, 08908 Spain; 2 Faculty of Nursing, University of Barcelona, S/N Feixa LLarga, Pavelló de Govern 3a Planta, Barcelona, L’Hospitalet de Llobregat, 08907 Spain

**Correction: BMC Palliat Care 23, 41 (2024)**.

10.1186/s12904-024-01359-w.

Following publication of the original article [1], the author reported that they missed to include the following statement in the acknowledgement section: **We thank the CERCA Programme/Generalitat de Catalunya for their institutional support.**

The original article has been updated.
